# Knowledge and perceptions about Dolutegravir and Dolutegravir counselling: a qualitative study among women living with HIV

**DOI:** 10.1186/s12905-023-02630-7

**Published:** 2023-09-09

**Authors:** John C. Chapola, Fan Lee, Agatha Bula, Nora E. Rosenberg, Jennifer Tseka, Maganizo Chagomerana, Mina C. Hosseinipour, Jennifer Hui-Yu Tang

**Affiliations:** 1 University of North Carolina Project-Malawi, Lilongwe Private Bag A104, Lilongwe, Malawi; 2 Duke University, Durham, NC USA; 3 Gilling’s School of Global Public Health, University of North Carolina, Chapel Hill, NC USA; 4 Division of Infectious Diseases, School of Medicine, University of North Carolina, Chapel Hill, NC USA; 5 Department of Obstetrics and Gynecology, University of North Carolina, Chapel Hill, NC USA

**Keywords:** Dolutegravir, Efavirenz, Antiretroviral therapy, Adherence, Neural tube defects

## Abstract

**Introduction:**

In 2018, the Malawi Ministry of Health adopted the recommendation to switch first-line antiretroviral therapy (ART) from an efavirenz (EFV)-based to a dolutegravir (DTG)-based regimen. Little is known about patients’ experience during this transition. We conducted a qualitative study to explore DTG-related counselling challenges among providers of HIV care and factors influencing regimen switching or non-switching among women living with HIV in Lilongwe, Malawi.

**Methods:**

Between February-July 2020, we recruited participants who took part in DTG counselling on reasons to switch, side effects, and benefits from two government health facilities providing HIV care: Area 18 health centre and Bwaila district hospital in Lilongwe, Malawi. We purposively sampled and interviewed 8 women living with HIV who remained on an EFV-based regimen after counselling, 10 women who switched to a DTG-based regimen, and 10 HIV care providers who provided counselling about ART switching. In-depth interviews were used to explore patient’s perceptions of DTG, factors affecting the decision to switch, and both patient and provider experience with counselling. Interview data was coded for themes using inductive and deductive codes. Interviews were conducted until thematic saturation was achieved. Data matrices were used for analysis and thematic extraction.

**Results:**

Most women in both groups were well versed on DTG’s potential side effects and felt well counselled on the benefits of switching, such as quicker viral load suppression. Many women associated DTG with birth defects and expressed concern. However, the primary reason for not switching was concern with how the new medication would be tolerated, especially when they were satisfied with their current regimen. Almost all providers expressed difficulty providing DTG counselling. Primary reasons included feeling inadequately trained and/or not having resources to use during counselling, such as diagrams or brochures.

**Conclusion:**

DTG counselling was well accepted by women; however, some felt that their concerns were not fully addressed. Providers reflected this sentiment in that they did not feel adequately trained or well-equipped to provide adequate counselling. Training on counselling for new ART regimens should be intensified and utilize patient-centered educational materials to address the concerns raised by both patients and health care providers.

**Supplementary Information:**

The online version contains supplementary material available at 10.1186/s12905-023-02630-7.

## Introduction

Efavirenz (EFV)-based antiretroviral therapy (ART) has been the first-line treatment for HIV-1 in Malawi since 2011, including for women receiving ART in pregnancy. However, Dolutegravir (DTG), an integrase strand transfer inhibitor (INSTI), has shown superior efficacy and tolerability compared to non-nucleoside reverse transcriptase inhibitors (NNRTIs), such as EFV [1]. DTG-based ART regimens have shown clear advantages over EFV-based regimens in improved viral suppression, tolerability, and higher genetic barriers to HIV-1 resistance. Furthermore, the DTG-based regimen of Tenofovir, Lamivudine and DTG (TDF/3TC/DTG) is available as a generic in low- and middle-income countries (LMICs) at a median price of US $75 per person-year, which is more affordable than the first-line EFV-containing regimen [2] [3]. In light of these advantages, in 2018, a formal transition from EFV-based first-line regimen to DTG-based first-line regimen was initiated in many countries, [4] including Malawi.

The Malawi Ministry of Health (MoH) initially announced the adoption of DTG-based pill as its first-line regimen for men of all ages and women over the age of 45 years [5]. Women under 45 years old were excluded due to concerning preliminary results from a birth defect surveillance study (TSEPAMO) in Botswana, which showed increased risk of neural tube defects (NTDs) among women taking DTG [5]. However, new evidence from additional clinical trials in Africa showed that the risk of neural tube defects is substantially lower than what was initially seen, and while the risk of birth defects is of concern, the benefit of DTG in elimination of mother-to-child HIV transmission (eMTCT) and long-term maternal health was substantial [6]. Therefore, in 2019 the World Health Organization (WHO) recommended DTG as the preferred first line ART for all persons living with HIV, including women of childbearing age and pregnant women.

Following these recommendations, the MoH announced that Malawi would accelerate initiation of DTG-based regimens for newly diagnosed Malawians living with HIV with weight over 20 kg and allow women of reproductive age to switch to the more effective DTG-based regimen, based on an informed choice. The MOH also stipulated that although access to long acting contraception should be supported for women living with HIV who are of reproductive age and are on ART, women were not required to use contraception when opting for DTG [7] [8].

However, regimen change can affect medication adherence, especially in the setting of potential fetal concerns of DTG-based ART. Poor counselling about new medications or medication switching can lead to negative psychological factors that affect adherence, such as perceived inefficacy and stigma due to misunderstanding. The Malawi MoH initiated a training program for counselling patients on the DTG-based regimen. Through the Department of HIV & AIDS and Viral Hepatitis (DHA), the MoH trained all the ART providers across the country, which included nurses, clinicians, Health Surveillance Assistants (HSAs), and HIV Diagnostic Assistants (HDAs) on DTG. Classroom presentations about DTG were held, and practical sessions about DTG counselling were performed before proceeding with implementation.

However, little is known about how Malawian women living with HIV (WLWH) responded to the counselling about the potential risks and benefits of switching to DTG-based ART or factors that affected their decision making on whether or not to switch. Understanding patient experiences with DTG counselling and motivational factors for medication preferences is important in the successful rollout of national first-line ART switching. Furthermore, understanding the challenges HIV providers experienced in counselling on ART switching can provide insight into effective program implementation. No study to date has evaluated the perspectives of female patients or HIV providers in Malawi regarding the DTG switch campaign. We therefore conducted a qualitative study to describe patient experiences with counselling about switching from EFV-based to DTG-based therapy, understand factors important for WLWH’s decision making on ART switching, and explore provider perspectives on DTG counselling.

## Methods

### Study population

This exploratory qualitative study employed in-depth interviews (IDIs) to understand knowledge and perceptions about DTG counselling among WLWH who received DTG counselling (patient-participants) and providers who administered DTG counselling from 2018 to 2019 (provider-participants) in two facilities in Lilongwe District, Malawi.

Patient-participants of this study were women who underwent DTG counselling and were recruited from two different groups. The first group was from participants of an ongoing study called S4, which focused on HIV Prevention of Mother-to-Child Transmission (PMTCT). The study was conducted by UNC-Project Malawi, a biomedical research institution in Lilongwe, Malawi, which is a collaboration between the University of North Carolina at Chapel Hill (UNC) and the Malawi MoH. The aim of the S4 study was to characterize safety, durability, ART resistance, and clinical outcomes for mothers and infants exposed to EFV-and Atazanavir/ritonavir-based ART regimens. Participants of S4 were followed for three years after enrollment and received their HIV care at two locations: (1) Area 18 health center, a community health facility that provides outpatient care including HIV care, and (2) The antenatal clinic and family planning clinic at Bwaila hospital, a district facility that provides both outpatient and inpatient care. The second group of women recruited as patient-participants were those receiving non-S4 related care at the Area 18 or Bwaila ART clinics, which are run by the Malawi Department of HIV & AIDS and Viral Hepatitis a department within the Malawi Ministry of Health.

### Counselling protocol

The switch from an EFV-based regimen to a DTG-based regimen was being offered at both Area 18 and Bwaila. Study nurses for the S4 study were trained using a UNC-Project and Lighthouse Trust-developed counselling script and performed DTG counselling with S4 participants from the 9-month visit to the 36-month visit. The counselling script was derived from WHO guidelines on DTG and included information on important advantages, contraindications, and side effects of DTG. S4 study organised study trainings where study nurses were briefed about DTG, following WHO and MoH guidelines. The study nurses were also trained on how to provide DTG counselling using the DTG counselling script. When the training was completed, dry runs and practice sessions were held before proceeding to their actual implementation. The Department of HIV & AIDS and Viral Hepatitis also trained all the ART providers across the country on DTG, which included nurses, clinicians, HDAs and HSAs. They held classroom presentations about DTG and practical sessions about DTG counselling before proceeding to actual implementation.

### Recruitment of participants

To assess the knowledge of WLWH about dolutegravir and their perceptions of DTG counselling, we aimed to recruit a total of 20 WLWH (10 from Area 18 health facility and 10 from Bwaila hospital) for in-depth interviews. We purposefully selected participants, with the goal of including 5 women from each site who remained on efavirenz (EFV) and 5 women from each site who switched to dolutegravir.

At the two facilities, the patient participants were recruited from either the S4 study or non-S4 related ART clinic. The inclusion of patient participants from the S4 study was motivated by the availability of routine counselling at the study clinics, which was sometimes unavailable in non-S4 related clinics.

Among the S4 cohort, we recruited women attending their 9- to 36-month follow-up visits who were within 2 weeks after receiving DTG counselling. Eligible women were identified through study’s enrolment logs and contacted via mobile phones. For those eligible and interested in participating, appointments for IDIs were scheduled at the clinic where they received care. A total of 8 women were sampled and all the women were eligible and interested to participate in the study. Among women receiving non-S4 related HIV care, we purposefully sampled 10 women who had just received DTG counselling, all the 10 women were eligible and interested, we collected their locator information and scheduled IDIs at the clinics where they received care.

For provider-participants, we aimed to enrol 10 ART providers, 5 in each clinic and at least 3 who were S4 providers. We requested names of Nurses, Clinicians, HDAs, and HIV counsellors involved in DTG counselling from health facility managers and used purposeful sampling. We approached providers in clinic for recruitment and for those eligible and interested, IDIs were scheduled in their respectively clinics.

Informed consent was obtained from all the participants prior to interviews. All IDIs were scheduled within 2 weeks from DTG counselling and conducted in a private room allocated to the RA at each facility. IDIs took approximately 35 min for patient-participants and 25 min for provider-participants. All recruitment and interviews took place between 25 February and 23 July 2020.

### Data collection

Our aim was to gain insights into the role of counselling in women’s decisions to either switch or maintain their current antiretroviral therapy (ART) regimen. The interviews focused on the patient participants’ perceptions of DTG and the quality of DTG counselling provided by health workers at each facility. The study used IDI guides with probing follow-up questions to enable the RA to pursue in-depth information around DTG and gain greater understanding of perceptions about DTG counselling. The IDIs were conducted by a locally recruited and trained research assistant (JT) fluent in Chichewa and English. The IDIs focused on three thematic areas: (1) Patient knowledge and perceptions of DTG, (2) The decision-making process and potential factors that affected patient preference to switch or not switch from their current ART regimen to a DTG-based ART regimen, and (3) Provider facilitators and barriers for counselling women on DTG. Interviews were recorded, translated, and transcribed in English for analysis.

### Data analysis

Thematic analysis was used to analyse IDI data [9]. All transcripts were read until content became intimately familiar. Emergent themes were extracted from data review. A list of inductive codes was created and documented in a codebook based on identified themes, in addition to structural codes corresponding to initial interview questions in the IDI guide. An iterative coding process between two coders (JC & FL) was utilized using NVivo® version 12.0. The coded data was used to generate data matrices with summaries and quotations of each theme listed by participant [10].

## Results

### Demographic characteristics

We approached 18 WLWH (8 from the S4 study, and 10 from Area 18) and 10 health care service providers (5 from Area 18, and 5 from Bwaila). All were eligible, willing to participate and enrolled in the study. We were unable to reach our enrolment target for patient participants because the MOH changed its decision to offer women living with HIV a chance to decide whether to switch to DTG or continue with EFV ART. By mid-July 2020, DTG had been rolled out in all health facilities in Malawi which made most women ineligible for the study. The patient-participants ranged from 22 to 45 years old (Table 1: *Baseline characteristic of patient/client participants)*. Most women (12) had education up to primary level education, the majority (13) were married and had 1 or more living children. Most of the women had only been on EFV-based ART for almost 4 years (n = 12). Of the providers, the majority (6) were nurses, 2 were HDAs, 1 was an HIV counsellor, and 1 was a Clinical Officer. Among the 6 nurses, 3 were recruited from S4 study (1 from Area 18, 2 from Bwaila), and the other 3 were MoH nurses. The HDAs, HIV counsellor, and Clinical Officer were all MoH staff.

**Table 1:** Baseline characteristic of patient-participants (N = 18)

Characteristics	Number
Age	
Mean Age	30.6
Range	22–45
Level of education completed?	
No formal education	1
Some or completion of primary	12
Some or completion of secondary	5
Partner or no partner	
Partner	13
No partner	5
ART Duration by Years	
Mean	4.6
Range	2 − 1
Efavirenz Duration by Years	
Mean	4.1
Range	2–8
Number of Children Living Children	
0	1
1–2	8
3–4	7
5–6	2

### Client’s recollection of the counselling

Most women reported being counselled at least twice during their ART visits, with some counselled more than three times. Some reported being counselled once, one could not remember, one reported being counselled every visit and others reported not being counselled at all. The majority reported being counselled by a nurse, doctor, or Clinical Officer working at the ART clinic. We noted that health care providers who were not in the uniform attire worn by nurses were described generally as doctors or Clinical Officers by our study participants.

### Client’s knowledge and perceptions of DTG after counselling

Participants reported knowing the potential side effects of DTG, and references were commonly made regarding pregnancy and childbearing. For example, one woman explained that DTG causes headaches and excessive weight gain and is taken by people who have no desire to have children:

“This drug has a lot of issues, such as headaches and excessive weight gain. But I still insisted that they switch me…. I noted that the new regimen was better than the old, I thought it was better from what they told me…. When taking this drug, you must be on family planning. Be on a family planning method or you have no desire to give birth again…. it causes headaches and increases weight.” (EFV-IDI-B004).

Most women on DTG talked about the benefits of taking DTG, including quicker viral load suppression, the ability to take medication in the morning, and the drug’s ability to restore people’s strength quickly. For example, one woman explained that DTG gave her strength and kept her awake compared to her previous ART medication:

“So, this drug has just been manufactured, and it gives strength to a person because virus multiplication is reduced quicker in one’s body, and as it is taken in the morning, it removes drowsiness. You do not sleep. Because the old night drug, once you took you would feel sleepy right away.” (DTG-IDI-008).

Another participant explained:

“What I know about this drug is that they quickly reduce the virus in the body, and the immunity is high due to the decreased virus levels.” (DTG-IDI017).

However, almost all the women shared the perception that DTG was not meant for women of childbearing age, rather it was meant for men and older women. This was based on the initial communication by the MoH on who was eligible to take DTG based on the TSEPAMO study in Botswana. For example, one woman explained:

“It is only those who are older and male who are eligible take them, but those who are young and still in childbearing stage should still take the old ones, the evening ones [5A] …., these new drugs are for those who have stopped childbearing.” (EFV-IDI-A013).

Some of the women explained that DTG was designed as a family planning measure to reduce population growth:

“I thought maybe they just want us to stop giving birth, that is why they want to give us. Some people said people could still give birth, while others said they would not give birth. I just accepted whatever my fate would be.” (DTG-IDI-A007).

### Factors influencing the decision to switch to DTG-based regimen or stay on EFV-based regimen

Most of the women who switched from EFV to DTG explained that they switched because they were excited to take the medication in the morning and wanted to try the new medication. Providers recommended taking EFV at night because of some of the side effects associated EFV such as dizziness, drowsiness ad trouble concentrating. Taking medication in the morning benefited most women because they could not easily forget taking them. For example, one woman explained:

“I was just excited because at times I could fall asleep soon after eating without taking the medication while in the morning, as at now I take without any challenges.” (DTG-IDI-B003).

Another woman explained:

“I was excited because changing the drug was something of value to me because once we take this drug in the morning, and we just drink water only [no food] there would be no challenge. I accepted to taking this morning drug unlike the night ones.” (DTG-IDI-B006).

Others seem to be influenced by their partner’s experiences with DTG. One woman said:

“At the time I heard this, I had not yet started the new drug, but when I heard this, I felt like it was better not to switch and to stick to the old drug because of the risks they mentioned. Then when I came back and they explained again and having seen my partner take them same at home, and he shared the benefits, I felt it better for me to switch. Such that when I came here, I accepted to switch”. (EFV-IDI-A014)

The majority of the participants who switched responded that the decision to switch regimen was made by themselves, except for a few patients recruited from government health facilities who responded that the decision was made for them by health workers.

“No! I made the decision myself; the nurse just explained to me that there was a new drug. I was the one who said they should give me the new drug.” (DTG-IDI-B003).

Another participant explained:

“No. They just said we have switched your drug, and you should be taking this in the morning, then she gave me the drugs [DTG-based regimen] … So, it was hard to think about it, and initially I was refusing to be switched, I opted to keep on taking the evening ones [EFV-based regimen].” (DTG-IDI-A012).

Many of the women who refused to switch did so because they wanted to have children, and they reported that the medication was not meant for women of childbearing age.

“I decided that I should not take that drug, they said it was for those who are no longer in the childbearing stage, I was still having children. It was better for me not to take those drugs. That is why I take the same old ones.” (EFV-IDI-A015).

Others were waiting for their viral load results before they could be switched.

“they said it depends on my viral load, so maybe they noted that my immunity was low…. My viral load was high…. I was convinced to switch and be part of those taking this DTG. My immunity was low” (EFV-IDI-A014).

Some of the women were afraid of the potential new side effects of DTG and that affected their decision to switch.

“I was thinking about…I was just afraid for my life. That perhaps if I…I would ask myself what reactions I would have in my body, would I be ok, what life would I live compared to the old drug which my body was already accustomed to? That is why I had fears and concerns.” (EFV-IDI-A018).

Most of the women who refused to switch reported that the decision not to switch was made by themselves.

“I did it myself after I had heard about the new drug here at the clinic, so I decided to continue taking the old drugs [5A], he (her husband) did not even know what I did” EFV-IDI-A013.

Despite not having switched, most of the participants on EFV responded that, they would still consider switching to DTG based on the counselling they received at the clinic about DTG.

“The doctors provided the information, and I inherently made the decision…. we heard that the medication does not cause any problems, anyone can take them, those who want to have more children and those breastfeeding can take them…. So, I would like to try the new drug so that I too have my viral load suppressed” (EFV-IDI-A011).

### Facilitators and barriers for counselling women on DTG

We talked to Nurses, HDAs, HIV counsellors and clinical officers, from the two health facilities to understand the barriers and facilitators to counselling women about DTG. Almost all the service providers reported that they had difficulties adapting DTG counselling to routine ART counselling. This service provider felt she was not adequately trained to provide the information her patients were asking about.

“…but as I explained earlier, some people have strong beliefs (about new interventions), our work becomes hard when you are asked, you cannot convincingly respond to questions where things are contradicting each other. These are some things that hinder us service providers.” (SPA-009).

Another service provider said:

“It was hard because at first the message that was given was that this DTG was for men only and those who will no longer have children. So, you know how hard it is to try and convince a person otherwise. Some would shout at us and say “you want to do research on us with drug” etc. So as health workers we are not supposed to get angry with our patients but to sit with them and clarify things. We would tell them that yes, initially the drug was for a few specific people, but with ongoing research things have changed” (SPA-006).

Some of the service providers expressed that as time progressed, they got better at explaining the risks and benefits of DTG to their patients.

“We did not have a lot of information, especially if we were explaining to the person for the first time, but on their subsequent visit, we would be able to explain properly.” (SPB-004).

The majority of the service providers reported that DTG counselling provided enough information to patients to aid their decision to switch or not, after weighing the benefits and effects of DTG and EFV.

“Women were able to have enough information, and they were able to differentiate between DTG and 5A [EFV-based regimen] because of the counselling.” (SPB-005).

Furthermore, another provider explained that the decision to switch among most women was also based on the size of the tablet, the ability to take the medication in the morning, no drug-drug interactions with contraceptive methods, and fewer side effects.

“Some are saying it’s because the tablet is smaller as compared to the EFV-based regimen, and that they are taking the pill in the morning, and that it is not interacting with the family planning method, and that they don’t have the side effects they were experiencing when they were taking the EFV-based regimen” (SPB-001).

Service providers also explained that some patients trust HIV service providers and are willing to do what the providers recommend.

“I can say that all the women I have met and referred, do not refuse. Maybe it is because they are at a stage where they need help, and if they are pregnant or desiring to have children in the future, it is like they don’t have a choice, they want to be assisted depending on their status found out on that day. So, there is no room for refusal, when you say you will give them 13A [DTG-based regimen?], they just agree. Whatever drug you offer, they will agree, they do not argue much, but they still ask questions, because they are supposed to know if what other people were saying about side effects was true or not, so we have to clarify.” (SPA-007).

Almost all the service providers agreed that training specific to the risks and benefits of DTG-based regimens was required to provide effective counselling. There was a desire among providers to be experts for their patients, and for that to happen more training was required. For example, this provider explained:

“I think us counsellors should be trained a lot, like I said earlier, so that when we counsel a person on DTG, we would really know what we are talking about. The person being counselled will know that we went to school and were trained. Not getting to an extent where a person asks you a question, and you fail to answer, and wait to consult others due to lack of information…” (SPA-005).

Another provider said:

“People who are working at ART, they are the ones who interact with the participants. We have clerks, Expert Clients, counsellors, including us, I can just say all those involved in HIV testing and counselling. They should know all these things clearly because any person who comes asks anyone in the department. It is demoralizing when you must refer the person to someone else, and the client does not respect you as much.” (SPA-008).

## Discussion

Our findings showed that women in both groups had knowledge of DTG’s potential side effects which influenced their perceptions of the medication. Women in both groups also reported that that they felt well counselled on the benefits of switching. Many women associated DTG with birth defects and expressed concern; however, a primary reason for not switching was concern with how the new medication would be tolerated, especially when they were satisfied with the current regimen. Almost all the providers felt unprepared to counsel women about DTG as lack of resources and proper training were identified as some of the barriers to DTG counselling.

Access to information about the treatment benefits and side effects of DTG was a key factor in women’s decision-making process on whether to switch their medication in this study. Most of the women in this study highlighted that the decision to switch was made by themselves, but that was dependent on how much they knew about the medication. This finding is consistent with other studies that revealed that patients will often seek more information before making decisions about their health [11].

Little information about health interventions sometimes gives room to negative perceptions, which mislead patients and can affect the acceptance and adoption of health interventions [12]. However, a study of WLWH receiving care from government facilities in Kenya found that patients trusted information that came directly from providers more than their peers or others in their community [13]. This is reinforced by how well health care providers in health facilities communicate information to their patients [13]. Our results show that most of the women living with HIV were provided with information about DTG through initial communication by the MoH staff [4]. It is possible that these women took that information seriously and used it in their decision-making. The information provided by the health care providers aided their decision on whether to switch to the DTG regimen. Furthermore, since counselling was conducted by either a nurse, HIV Diagnostic Assistant (HDA), HIV counsellors, or clinical officers, there were chances that a patient’s decision would be influenced by the person conducting the counselling. It should be noted that in Malawi, nurses interact with patients most of the time, which means they could exert a more significant impact on the patients’ decision-making process. Nevertheless, we learned through the interviews that counselling alone was not enough for some women. Women take into account what their peers or family members are saying, and some want to use others’ experiences to inform decisions on whether to accept or reject an intervention [14] [15]. A patient’s family member can be a valuable source of health information and can collaborate in making an accurate diagnosis and planning a treatment strategy [16]. Health care providers need to know and understand the experiences, perceptions and concerns shared by peers and family members of the patient to come up with solutions that can improve health interventions [17]. Furthermore, others have also recommended that priority setting committees and health policy makers should utilise patient experiences to inform decisions. While there is a strong practice of using clinical, epidemiologic, and economic evidence for these decisions, patient experience can provide important, relevant insight into the nature of patients’ need, the condition, and the treatment under consideration [18].

Some of the women in this study talked about how they felt the decisions to switch or not to switch to DTG were made for them. According to the Malawi Clinical Management of HIV in Children and Adults Guideline, patients on any other HIV regimen not listed in the guideline are encouraged to remain on their current regimen unless there is a specific indication to change [7]. Some of the women in this study were not happy with the government guidelines and felt the decision to switch was taken away from them. Initially, women who were of childbearing age were not switched to DTG because of the initial communication from MoH that only women past childbearing age would be switched to DTG [7]. Later, all women were allowed to switch to DTG if they wished to do so, provided all the information about the DTG was provided to them [8] [19]. Our results show that despite the MoH decision to roll out DTG to men and women of all age groups, health care providers made the decision to switch women from their current regimen based on their VL results. For example, one woman explained that health workers explained to her that her ability to switch to DTG will depend on her VL. She thought that maybe they had noted that her VL was too high, and she needed to have her VL supressed before she could be switched. Removing personal autonomy in making health decisions can impact adherence as some women may not agree with the decision that was made for them.

The women in this study also described how they felt about the potential of bearing children with neural tube defects. Some were afraid of having children with neural tube defects, while others were afraid of what DTG would do to their own bodies. These concerns raise questions on whether WLWH would adhere to DTG if they want to have children. A study on patient experiences of switching from EFV to DTG-based ART in Uganda showed that women of childbearing potential were also concerned about neural tube defects from DTG exposure and that substitution may confer new side effects, which calls for ongoing information sharing and ART adherence support [20].

Quality counselling and social support systems can improve health-promoting lifestyle and quality of life among women living with HIV [21] [22]. Provision of quality counselling is dependent on how well the individual providing the counselling was trained. The type and quality of training which a provider receives has an influence on how much a provider knows about the subject matter [23]. The training has an impact on how well the provider or counsellor delivers the counselling session. In our study, there were inconsistencies on how the providers were trained about DTG. Some were trained more systematically, while others were just briefed about DTG. UNC Project Malawi, Lighthouse Trust, and The Department of HIV & AIDS and Viral Hepatitis conducted training sessions for healthcare providers, with the expectation that these trained providers would subsequently train their colleagues in their respective health facilities. This approach has often been adopted for sustainability reasons, like how most trainings are conducted by NGOs and MOH in Malawi. However, it is possible that this approach contributed to the inconsistencies in the trainings. While some providers took the initiative to thoroughly train their colleagues, others merely provided briefings, resulting in misinformation and improper training. As a result, the lack of standardized training procedures had a negative impact on the overall quality of the training received by healthcare staff across different facilities. Furthermore, lack of training rendered the provider’s inability to answer some questions raised by a patient on the subject matter. The consensus among the health care providers was that they needed to know what they were talking about to deliver the counselling effectively. The health care providers recommended that decision makers should consider training staff members before introducing a new intervention.

The decision to roll out DTG was made by the donors and governments in LMIC based on the fact that its benefits outweighed the risks and that it is cheaper to produce compared to EFV [2] [3]. It is important to know that there was a push to change to integrase inhibitors well before the costs came out. Furthermore, the western settings had essentially all moved to integrase inhibitors as first-line due to improved efficacy and side effect profile well before WHO recommendations [24] [25]. Despite that, our study has shown that in Malawi, not all providers and counsellors felt adequately trained to give important counselling for their patients. This gap could have implications on ART adherence if new changes in ART medications are not appropriately rolled out.

In Malawi, most people have now switched to DTG, but the lessons from this work highlight the need to plan for adequate counselling of providers and patients when policy dictates medication changes. One potential limitation of this study is the timing of the IDIs. In this study, the IDIs were conducted two weeks after the counselling sessions, which may be considered a relatively long time. This time gap could potentially lead to recall bias as participants might struggle to accurately remember or may forget certain details discussed during the counselling sessions. The longer the interval between the two events, the higher the likelihood that participants’ recollections may be influenced by external factors or fade over time. To mitigate the impact of recall bias, future research could explore conducting IDIs at a more immediate time after counselling or implement strategies to reinforce participants’ memory and engagement during the interviews.

It is also important to acknowledge that the participants and healthcare providers involved in research activities, such as the S4 Study, while attending healthcare facilities, may not be fully representative of all patients and providers in Malawi. These individuals are engaged in an important research activity, which may lead them to possess distinct characteristics or motivations that set them apart from the broader population.

Additionally, it is possible that this group (S4 Study), being aware of the research context, could be more sensitive to counselling interventions compared to other groups. Their heightened awareness and involvement in research activities might influence their responses and behaviour during counselling sessions, potentially impacting the generalizability of the study findings to the wider population of patients and providers in Malawi. Therefore, caution should be exercised when extrapolating the results to the broader healthcare context. Nevertheless, there is need for implementation studies that examine different counselling strategies, for example, examining a digital or blended learning training or a video-based counselling session, evaluating positive versus negative consequences of framed messages, or offering provider job aids and patient materials.

### Electronic supplementary material

Below is the link to the electronic supplementary material.

Supplementary Material 1

## Data Availability

The datasets generated and/or analysed during the current study are not publicly available due to the commitments made during the ethics application and consenting process. During the ethics review and participant consent stages we provided explicit assurances to both the ethics boards and the study participants that dedicated efforts would be undertaken to uphold the privacy and confidentiality of the data and study records. Respecting these commitments, we are continuously safeguarding the data collected during the research. By maintaining the confidentiality of the data, we aim to uphold the trust placed in us by the research ethics boards and the individuals who participated in our study. However, it’s important to highlight that the data can be made available by reaching out to the corresponding author through a reasonable request.
