# Supplementary material for: Knowledge and perceptions about Dolutegravir and Dolutegravir counselling: a qualitative study among women living with HIV
Source: BMC Womens Health. 2023 Sep 9;23:478. doi: 10.1186/s12905-023-02630-7 (PMC10492391; doi:10.1186/s12905-023-02630-7)
Supplement: Supplementary file 1 — Supplementary Material 1 [file 12905_2023_2630_MOESM1_ESM.docx]

**INFORMATION CRIPT FOR DTG BASED ART REGIMENS (13A, 14A, 15A)**

Provide the following information about DTG

- 1. Important Advantages
     1. More potent: rapid viral suppression within weeks
     2. More durable: high drug resistance barrier
     3. Convenient: small tablet taken once per day in the morning
     4. Better tolerated: very few patients experience significant side effects
     5. Emerging scientific evidence shows that the benefits of DTG outweighs the risks.
     6. Fewer drug-interactions
     7. Preliminary data from a birth defect surveillance study in Botswana suggest a possibly increased, but still very low, risk of neural tube defects (NTDs) in children born to women receiving DTG based regimen in the peri-conceptional period.
  2. Contraindications
     1. Women with body weight of less than 20 kgs
     2. Uncontrolled diabetes
     3. Renal Failure: creatinine clearance of less than 30ml/min
     4. Severe liver damage
  3. Side effects
     1. Insomnia, headache, agitation
     2. Nausea
     3. Skin rash
  4. Patient should always remember the following
     1. Always remember to take remaining pills when coming to the clinic
        1. Bactrim
        2. TB prophylaxis drugs
        3. Any vitamins
        4. Any BP drugs
     2. All women of reproductive age can opt for the more effective DGT based regimens based on an informed choice.
     3. Access to long-term acting contraception should be supported for HIV positive women of reproductive age on ART.
     4. Women are not encouraged to use short term methods of contraceptives when opting for DTG.
